# Exploring the Application Capability of ChatGPT as an Instructor in Skills Education for Dental Medical Students: Randomized Controlled Trial

**DOI:** 10.2196/68538

**Published:** 2025-05-27

**Authors:** Siyu Huang, Chang Wen, Xueying Bai, Sihong Li, Shuining Wang, Xiaoxuan Wang, Dong Yang

**Affiliations:** 1 State Key Laboratory of Oral & Maxillofacial Reconstruction and Regeneration, Key Laboratory of Oral Biomedicine Ministry of Education, Hubei Key Laboratory of Stomatology School & Hospital of Stomatology Wuhan University Wuhan China; 2 Center for Orthodontics and Pediatric Dentistry at Optics Valley Branch School & Hospital of Stomatology Wuhan University Wuhan China; 3 Department of Periodontology School & Hospital of Stomatology Wuhan University Wuhan China

**Keywords:** ChatGPT, dental education, clinical skills, artificial intelligence, randomized controlled trial, virtual reality, cognitive load, self-efficacy, motivation, spatial ability

## Abstract

**Background:**

Clinical operative skills training is a critical component of preclinical education for dental students. Although technology-assisted instruction, such as virtual reality and simulators, is increasingly being integrated, direct guidance from instructors remains the cornerstone of skill development. ChatGPT, an advanced conversational artificial intelligence model developed by OpenAI, is gradually being used in medical education.

**Objective:**

This study aimed to compare the effects of ChatGPT-assisted skill learning on performance, cognitive load, self-efficacy, learning motivation, and spatial ability, with the aim of evaluating the potential of ChatGPT in clinical operative skills education.

**Methods:**

In this study, 187 undergraduate dental students recruited from a first-class university in China were randomly divided into a ChatGPT group and a blank control group. Among them, the control group used videos for skill acquisition, and the ChatGPT group used ChatGPT in addition to the videos. After 1 week of intervention, skills were tested using desktop virtual reality, and cognitive load was measured by recording changes in pupil diameter with an eye tracker. In addition, a spatial ability test was administered to analyze the effect of ChatGPT on those with different spatial abilities. Finally, a questionnaire was also used to assess cognitive load and self-efficacy during the learning process.

**Results:**

A total of 192 dental undergraduates from a top-tier Chinese university were initially recruited for the experiment by October 25, 2024. Following eye-tracking calibration procedures, 5 participants were excluded, resulting in 187 eligible students successfully completing the experimental protocol by November 2, 2024. Following a short-term intervention administered through randomized allocation, superior performance (ChatGPT group: mean 73.12, SD 10.06; control group: mean 65.54, SD 12.48; *P*<.001) was observed among participants in the ChatGPT group, along with higher levels of self-efficacy (*P*=.04) and learning motivation (*P*=.02). In addition, cognitive load was lower in the ChatGPT group according to eye-tracking measures (ChatGPT group: mean 0.137, SD 0.036; control group: mean 0.312, SD 0.032; *P*<.001). The analysis of the learning performance of participants with different spatial abilities in the 2 modalities showed that compared to the learners with high spatial abilities (ChatGPT group: mean 76.58, SD 9.23; control group: mean 73.89, SD 11.75; *P*=.22), those with low spatial abilities (ChatGPT group: mean 70.20, SD 10.71; control group: mean 55.41, SD 13.31; *P*<.001) were more positively influenced by ChatGPT.

**Conclusions:**

ChatGPT has performed outstandingly in assisting dental skill learning, and the study supports the integration of ChatGPT into skills teaching and provides new ideas for modernizing skill teaching.

**Trial Registration:**

ClinicalTrials.gov NCT06942130；https://clinicaltrials.gov/study/NCT06942130

## Introduction

ChatGPT, developed by OpenAI, is a powerful artificial intelligence (AI) language model based on the GPT architecture designed to generate human-like text and engage in conversation [1-3]. It uses advanced deep learning techniques to understand various cues and respond with contextually relevant and coherent language [4,5]. Since its release, ChatGPT has been well received and multiple applications have been developed that integrate its chatbot capabilities. Many studies have reported on the potential of ChatGPT for passing examinations [1], learning anatomy, and understanding emerging trends [6,7], making it an important tool in areas such as medical education.

The core of skills education is to enable students to master clinical skills, such as dental restoration, periodontal treatment, and surgical procedures, through hands-on practice [8]. This hands-on approach allows students to apply theoretical knowledge to real cases [9]. Before engaging in actual clinical practice, students typically undergo training in simulated environments using traditional dental mannequins [10], 3D-printed models [11,12], and virtual reality (VR) technologies [13,14]. This training provides a risk-free setting where students can practice repeatedly until they achieve proficiency. Given the varying learning paces and skill levels among students, skills education often involves small-group teaching or one-on-one mentoring. Instructors tailor their guidance to students’ individual needs to ensure that each one attains the necessary clinical competence. However, this approach demands significant instructional resources.

Research has shown that ChatGPT can assist students in reviewing key concepts, reinforcing theoretical knowledge [1,15], and simulating clinical scenarios to enhance clinical reasoning [16]. This raises the question whether ChatGPT could also serve as an instructor in skills training. We found that the potential of ChatGPT for dental kills education remains unknown.

In addition, given the limited working space in oral procedures, fine motor skills and high spatial ability are crucial for mastering technical skills [17,18]. Instructional practitioners also need to incorporate Cognitive Load Theory [19,20] and the Control Value Theory of Achievement Emotions [21] into their instructional design; the former emphasizes minimizing the extraneous cognitive load, providing appropriate learning content for a given learner, and sparing sufficient working memory capacity for the germane cognitive load [22-26]. The latter highlights the importance of emotions which includes self-efficacy and motivation in academic achievements [27-29].

In this randomized controlled trial, we integrated ChatGPT into skill education and subsequently assessed the effectiveness of skill acquisition using high-fidelity desktop VR simulations. The investigation further evaluated the impacts of spatial ability, cognitive load, learning motivation, and self-efficacy. Hence, this study aims to investigate the potential value-added effects of a ChatGPT-integrated pedagogical framework on operative skill training in dental education, thereby providing evidence-based foundations for innovating dental education systems in the artificial intelligence era.

## Methods

### Recruitment

This study recruited 192 dentistry students (female: n=98, 51.04%; male: n=94, 48.96%) from a first-class university. In China, the dental medicine curriculum during the first 3 years of university focuses on foundational medical sciences and basic clinical medicine, with the aim of progressively developing clinical reasoning skills. In the fourth and fifth years, students transition to systematic instruction encompassing both theoretical knowledge and clinical skills in core dental disciplines. Therefore, participants in the study were in their fourth or fifth years of a 5-year dental education program and had received theoretical knowledge in courses. They ranged in age from 20 to 25 years, with an average age of 22.53 (SD 1.47) years. All participants’ visual acuity was normal or had been corrected to be normal.

### Procedure

Before the study, participants were calibrated for eye tracking using the 9-point method, and those who did not meet the calibration standards were excluded. Participants who met the inclusion criteria were randomly assigned to a ChatGPT-3.5–assisted learning group and a blank control group using the sealed envelope method to minimize systematic bias. The control group used videos for skill acquisition, while the ChatGPT group supplemented video learning with ChatGPT-3.5 as an additional learning tool. Following the completion of pretest questionnaires assessing theoretical knowledge, spatial ability, motivation, and self-efficacy, participants began a 1-week skill acquisition period. Subsequently, both groups were assessed on their skills using desktop VR (Zhonghui), and eye movement data were collected using an aSee eye-tracking device (EVERLOYAL). Data collection was conducted in a digital classroom with illumination levels maintained between 100 and 130 lux. Participants’ motivation and self-efficacy were also recorded.

### ChatGPT

**Figure 1:**
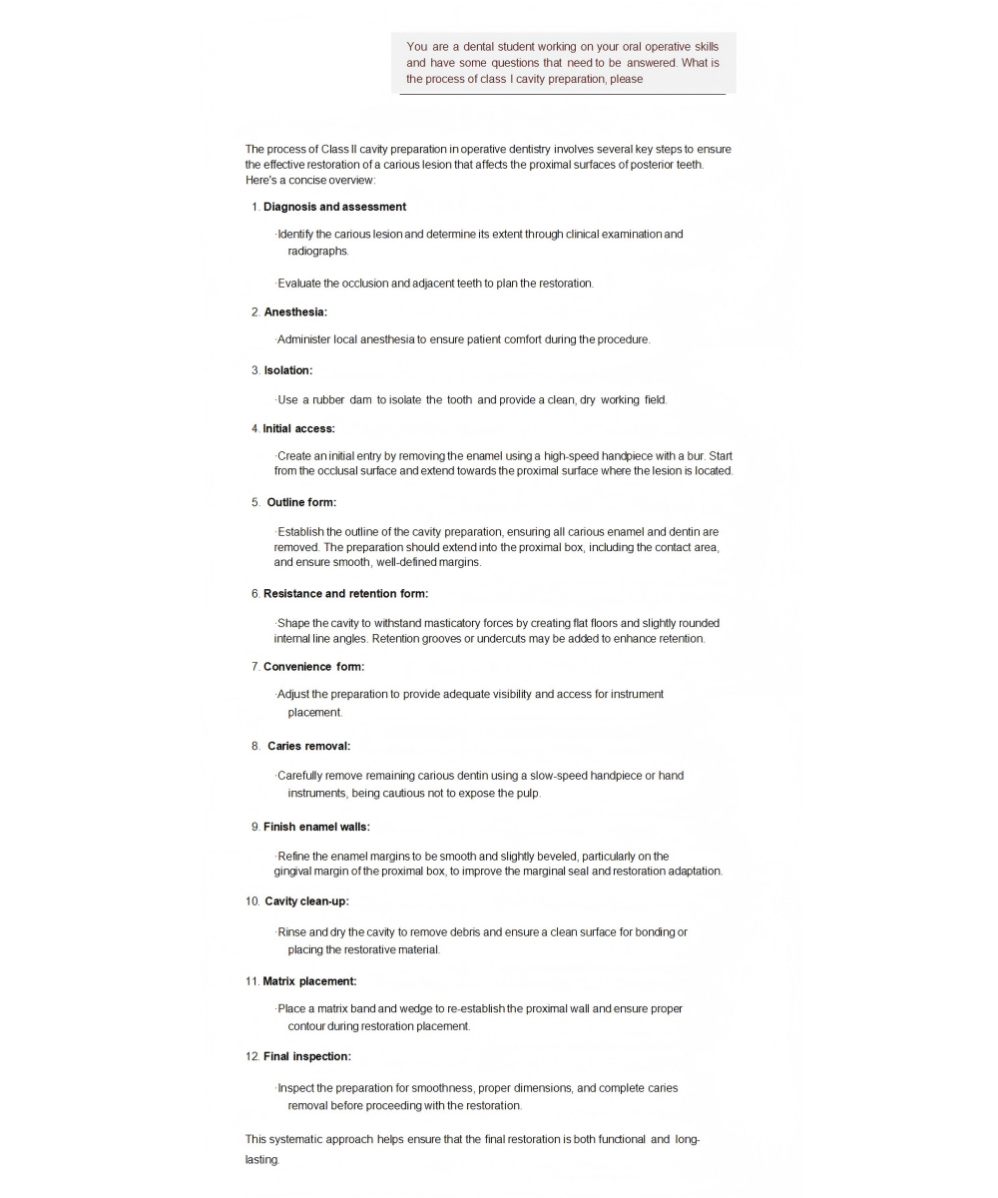
ChatGPT interface.

Although ChatGPT version 4.0 offers advantages such as enhanced comprehension, reasoning, and accuracy compared with version 3.5, the free version 3.5 (Figure 1) was used in this experiment due to its cost. Given that a recent study indicates nearly all faculty and students in Chinese universities have previous experience with generative AI tools, this study did not assess participants’ previous exposure to ChatGPT [30]. However, standardized operational guidelines were distributed to the ChatGPT group via group messaging platforms before the study to ensure familiarity and proficiency with the technology. During the skill learning period, the ChatGPT group was allowed to use ChatGPT to address doubts that arose during the operation process, verify the correctness of the procedures, and provide additional guidance as needed (Multimedia Appendix 1).

### Eye-Tracking Equipment and Software for Detecting Cognitive Load

Eye movements were monitored using the aSee eye-tracker (Multimedia Appendix 2) at a sampling rate of 60 Hz, tracking both eyes. Before the experiment, the participant’s position was calibrated to ensure that the distance between the screens and eyes was approximately 70 cm and participants were told they could move their head freely but not too much during the experiment. aSee eye-tracker was applied to collect and analyze the eye movement data.

### Theoretical Knowledge Test

A comprehensive knowledge test was formulated to assess participants’ theoretical understanding of the surgery (Multimedia Appendix 3). The test comprised 10 multiple-choice questions, and the content validity of these questions had been rigorously examined by experts, ensuring the relevance and appropriateness of the test content, and experts recommended the test be completed in 10 minutes. With each question scored out of 10, the total test score ranges from 0 to 100.

### Operational Test

**Figure 2:**
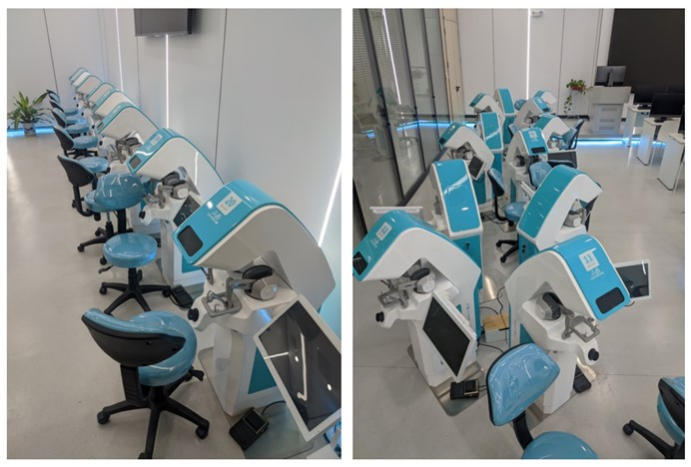
Virtual reality equipment.

At the end of the experiments, participants were asked to complete an operational test in desktop VR within 15 minutes (Figure 2). Operational test scores are automatically generated by the VR, which avoids the influence of subjective factors on test results.

### Spatial Ability Test

#### The Purdue Spatial Visualization Test

The Purdue Spatial Visualization Test: Rotations, developed by Roland Guay, was used to gauge the spatial ability of participants. It consists of 30 questions designed to assess participants’ ability to mentally rotate a 3D object. Guay recommended a time limit of 20 minutes to complete the test. The total score is 30 points with 1 point per question. Participants were then divided into groups based on their spatial ability, using the median as a distinction between those with high spatial ability and those with low spatial ability.

### Learning Motivation and Self-Efficacy

To assess students’ learning motivation and self-efficacy, this study used questionnaires that have been widely investigated in dental, medical, and nursing practice. The questionnaire contains 5 items focusing on the motivation domain and 5 items focusing on self-efficacy. Each item was coded according to a 5-point Likert rating scale (1=“strongly disagree,” 2=“disagree,” 3=“neutral,” 4=“agree,” and 5=“strongly agree”). The corresponding Cronbach α values were 0.76 and 0.73, indicating satisfactory internal consistency. Validity was censored by experts in medical education. All participants were requested to complete the questionnaire both before and after the experiment, with a time allocation of 10 minutes for this task.

### Statistical Analysis

**Figure 3:**
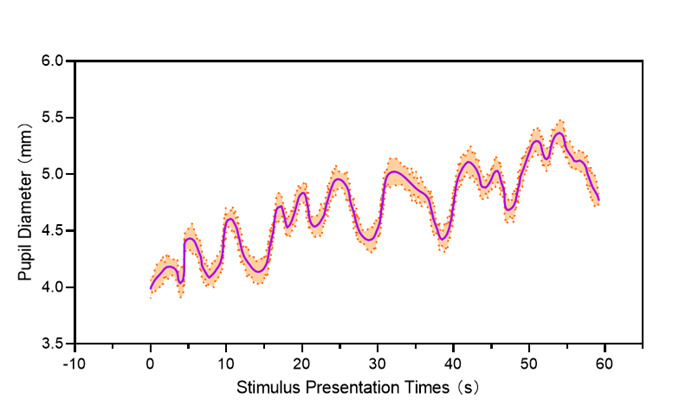
Smoothed pupil diameter.

An independent samples *t* test was used to determine the difference in performance and spatial ability, while the Mann-Whitney *U* test was used to compare the scores of self-efficacy and learning motivation. The raw pupil diameter data were extracted to measure cognitive load. For further signal processing, data points labeled as a blink and 50 milliseconds before and after these points were removed since eyelid movement during these periods might distort pupil diameter. MATLAB programming (MathWorks) was used to eliminate the fluctuations in pupil size to obtain a smooth curve of pupil size change over time, as depicted in Figure 3. In the analysis of pupil diameter variations during learning, this study calculated the median pupil sizes at the beginning of learning which was marked as the baseline, and the overall median pupil sizes during the learning process separately. The preference for the median over the mean was driven by the former’s greater robustness toward noise and outliers.

### Ethical Considerations

The trial protocol followed the CONSORT-EHEALTH (Consolidated Standards of Reporting Trials of Electronic and Mobile Health Applications and Online Telehealth; version 1.6.1) checklist (Multimedia Appendix 4) [31]. Informed consent was obtained from all participants and the study was approved by the ethics committee of the School and Hospital of Stomatology, Wuhan University (WDKQ2024-034) before it was conducted.

## Results

### Overview

The research results include the distribution of participants, knowledge test scores, operational test scores, and change in pupil diameter, which is used to measure the cognitive load, spatial ability test, learning motivation, and self-efficacy.

### Distribution of Participants

**Figure 4:**
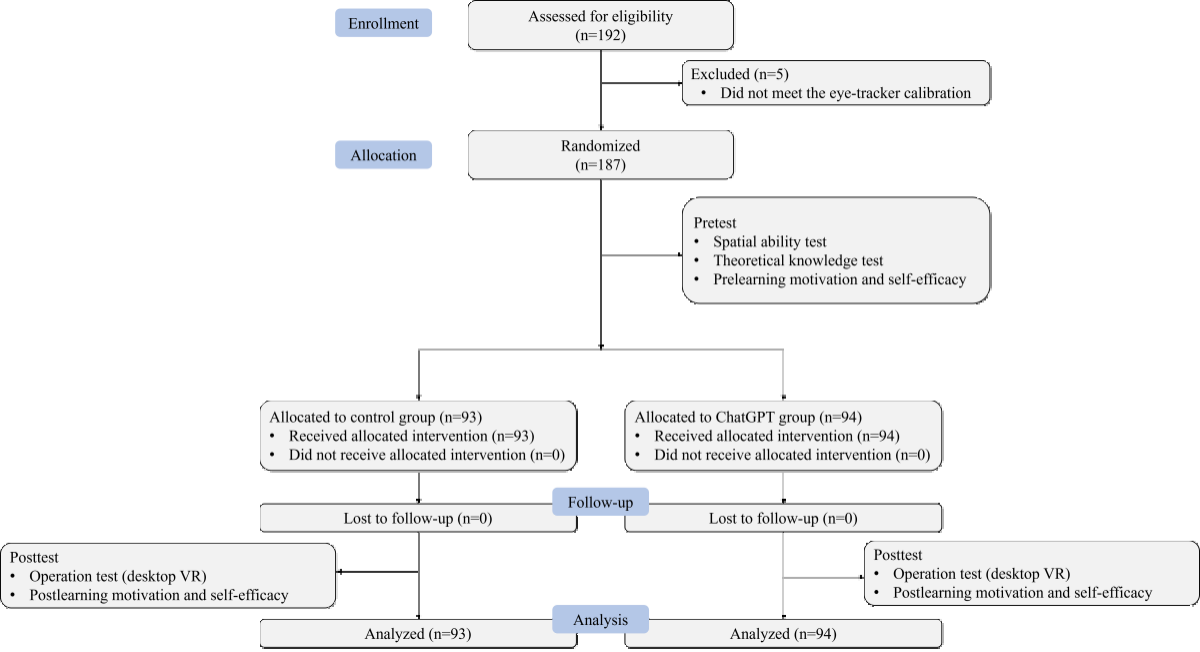
CONSORT (Consolidated Standards of Reporting Trials) flow diagram. VR: virtual reality.

During the eye-tracking calibration, 5 participants were excluded because they did not pass through the calibration. Thus, during the eye-tracking calibration, 94 students were randomly assigned to the ChatGPT group and 93 to the control group. The sex distribution was balanced, with 50.80% of female participants (26.2% in the ChatGPT group and 24.6% in the control group) and 49.20% of male participants (24.06% in ChatGPT the group and 25.13% in the control group), ensuring a representative sample. The detailed experimental procedure is illustrated in Figure 4.

### Theoretical Knowledge Test and Operational Test Scores

This paper presents a comparative analysis between the ChatGPT group and the blank control group in terms of theoretical knowledge and operational performance. Shapiro-Wilk tests were used to assess the normality of the data, which showed that the data in both groups followed a normal distribution. Due to the normality, *t* tests were performed to determine significant differences.

The results (Table 1) showed that there was no statistically significant difference in the theoretical knowledge test scores between the 2 groups (*t*_185_=0.649, *P*=.52), suggesting that the participants’ knowledge levels before learning operations in the 2 groups were comparable. However, the *t* test for operation performance revealed a statistically significant difference (*t*_176.241_=4.569, *P*<.001), indicating that the learning modes exerted an impact on operation learning. Specifically, the ChatGPT group exhibited a significant advantage over the video-only group in the operation training. It is noteworthy that all statistical tests were conducted at the α=.05 significance.

**Table 1:** The comparisons of 2 tests between ChatGPT and the control group.

Test	Knowledge test score, mean (SD)		*t* test (*df*)	*P* value
	ChatGPT	Control		
Theoretical	41.24 (8.19)	42.03 (8.34)	0.649 (185)	.52
Operation	73.12 (10.06)	65.54 (12.48)	4.569 (176.241)	<.001

### Cognitive Load

**Figure 5:**
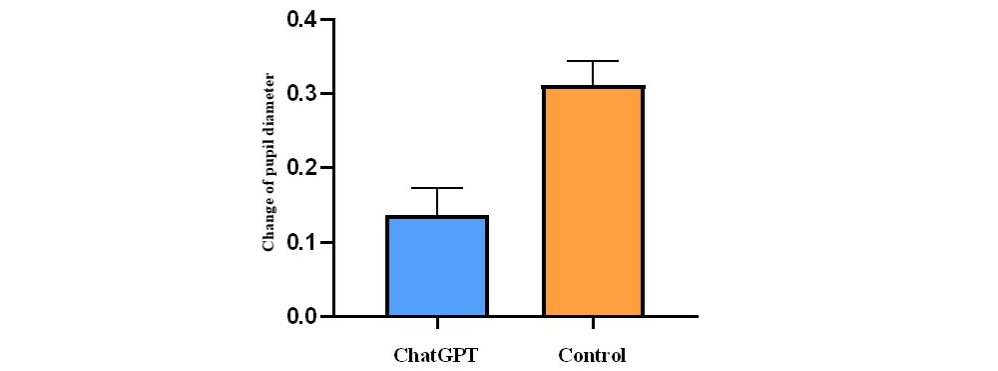
Comparison between 2 groups in the change of pupil diameter.

To access and compare the cognitive load experienced by the participants during the learning, changes in the participants’ pupil diameters were analyzed. Preliminary screening of the data confirmed that they followed a normal distribution. Figure 5 shows the mean increase in pupil diameter from baseline. An independent samples *t* test was performed to determine the statistical significance of the changes in pupil size. The results of the *t* test (*P*<.001), performed at a significance level of α=.05, showed significant differences between the ChatGPT group (mean 0.137, SD 0.036) and the control group (mean 0.312, SD 0.032). This implies that the cognitive load borne by participants in the ChatGPT group was significantly lower than that in the control group.

### Influence of Spatial Ability Based on Different Learning Modes

In order to categorize the participants according to their spatial abilities, the median was taken as the boundary. Table 2 provides an insight into the distribution and operational performance of participants with varying spatial abilities. In terms of the theoretical knowledge test, no statistically significant differences were found between the two modes, irrespective of whether participants belonged to high (*t*_92_=0.689, *P*=.49) or low (*t*_91_=0.764, *P*=.45) spatial ability groups. The independent samples *t* test, as presented in Table 2, indicated that there was no compelling evidence to support the notion that learning mode had a different impact on high spatial ability learners, However, for low spatial ability learners, the *t* test results indicated a statistically significant impact of the learning mode. With regard to those with low spatial abilities, the operational scores achieved by the ChatGPT group (mean 70.20, SD 10.71) were found to be higher than those attained by the control group (mean 55.41, SD 13.31).

**Table 2:** Distribution and t test for operational performance in different learning modes among high and low spatial ability learners.

Spatial ability and mode		Participants, n	Operational performance score, mean (SD)	*t* test (*df*)		*P* value	
**High**					1.23 (92)		.22
	ChatGPT	43	76.58 (9.23)				
	Control	51	73.89 (11.75)				
**Low**					5.94 (91)		＜.001
	ChatGPT	51	70.20 (10.71)				
	Control	42	55.41 (13.31)				

### Learning Motivation and Self-Efficacy

**Figure 6:**
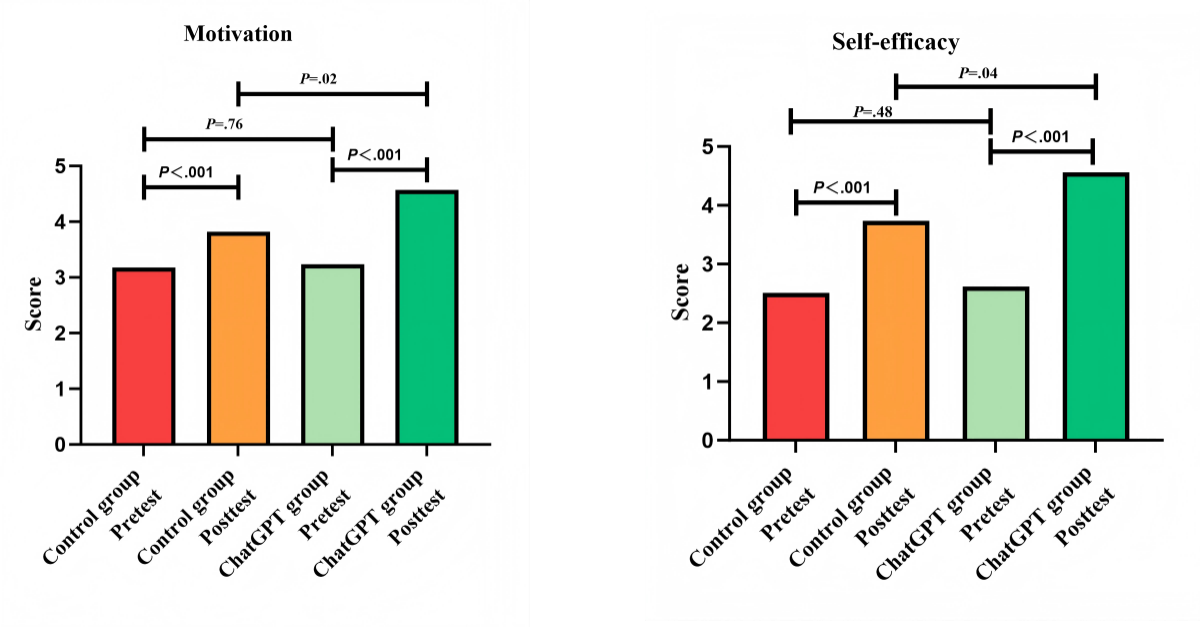
Motivation and self-efficacy pretest and posttest scores.

Given that learning motivation and self-efficacy scores were not normally distributed, the Mann-Whitney *U* test was used to analyze the differences between the pretest and posttest scores for ChatGPT and the control group respectively. Furthermore, this study further compared the scores between the 2 groups. Figure 6 demonstrates that, at the outset of the study, there was no evidence to suggest that there were any differences in learning motivation (Z=0.31, *P*=.76) and self-efficacy (Z=0.69, *P*=.48) between the 2 groups before the intervention. Nevertheless, it is notable that the posttest scores for learning motivation and self-efficacy differed from the pretest scores, irrespective of whether the learners were in the ChatGPT or control group. Furthermore, in comparison to the control group, the ChatGPT group exhibited higher posttest scores for both learning motivation (Z=2.32, *P*=.02) and self-efficacy (Z=2.03, *P*=.04).

## Discussion

### Principal Findings

The study revealed that incorporating ChatGPT-3.5 as a tutor resulted in markedly elevated performance scores in skill assessments compared with using videos in isolation. This discrepancy was particularly pronounced among students with lower spatial abilities. In addition, the use of ChatGPT-3.5 was shown to reduce cognitive load, enhance self-efficacy, and boost learning motivation. These findings provide robust evidence supporting the use of ChatGPT-3.5 as a valuable tool in skill training and offer insights for the design of educational programs.

Our investigation builds on previous studies that have identified the potential of ChatGPT in medical education. A recent study demonstrated that ChatGPT exhibited exemplary performance in both the immediate and long-term contexts of orthopedic teaching for undergraduate students [1]. In the field of dental education, extant research indicates that ChatGPT demonstrates satisfactory performance across a range of dental assessment types [32,33]. However, these studies have primarily focused on theoretical knowledge. Our findings further reveal that ChatGPT exhibits comparable exciting potential in skills-based education. Compared with traditional dental education, ChatGPT offers personalized learning content and feedback tailored to individual student needs, helping students acquire skills more effectively. In addition, ChatGPT provides real-time feedback and answers to student queries, thereby enhancing the efficiency of the learning process [34]. It also alleviates the strain on teaching resources and offers greater flexibility in skill training schedules.

Changes in pupil diameter during task performance have been used to infer variations in cognitive load. A previous study applied this method to measure cognitive load differences between experts and students while examining dental radiographs [35]. In line with this approach, this study used eye-tracking technology to record pupil diameter changes from baseline. The results indicated that participants in the ChatGPT-assisted group exhibited smaller changes in pupil diameter, suggesting a reduction in cognitive load. This decrease can be attributed to ChatGPT’s ability to address challenging points in the skill-learning process. These findings not only validate the efficacy of ChatGPT in reducing cognitive load but also highlight its potential as a pedagogical tool that fosters a smoother and more relaxed learning experience.

Emerging evidence indicates the varied performance of AI models in spatial relations–related topics. Previous studies evaluating ChatGPT on Geographic Information Systems examinations demonstrated its capacity to achieve passing scores in spatial analysis, spatial statistics, and interpolation tasks [36]. Furthermore, generative AI systems including ChatGPT-3.5 have shown nascent potential in executing basic spatial queries [37]. However, limitations persist, as evidenced by its suboptimal performance in robot programming scenarios requiring complex 3D spatial reasoning and nuanced understanding of spatial relationships [38]. Nevertheless, there is no research on the topic of the spatial relations related topics between ChatGPT and medical skill learning, and this study provides a preliminary exploration of this component.

Previous research has demonstrated a significant yet modest positive correlation between spatial ability measured by the Purdue Spatial Visualization Test: Rotations and performance in dental anatomy assessments [39]. Furthermore, studies using other spatial ability assessments, such as the mental rotations test, visualization of views test, and visualization of rotation test, have consistently shown that students with higher spatial aptitude achieve superior performance in endodontics [40], radiology [41], anatomy [42], and prosthodontics [43]. The convergent validity of these findings across diverse spatial ability metrics and dental subdisciplines suggests that spatial ability may serve as a foundational competency in skill acquisition. This study delves into the impact of spatial ability on learning outcomes under different instructional modes, revealing the interaction between spatial ability and teaching methods. The findings indicate that ChatGPT-assisted instruction significantly enhances learning outcomes for learners with lower spatial ability. However, no significant difference was observed between the 2 instructional modes for learners with higher spatial ability. Cognitive load theory provides a framework for understanding these results. Given the limited capacity of working memory to process information simultaneously, learners with lower spatial ability in the control group experienced cognitive load beyond their cognitive resources [20]. In contrast, learners with higher spatial ability could activate preconstructed schemas based on 2D images, thus reducing the demand on working memory [20,44]. ChatGPT, by offering clear explanations and guidance, helps learners with lower spatial ability better comprehend complex skills or concepts, thereby alleviating cognitive load and improving their learning outcomes.

Besides, the findings of this study indicate that participants in both learning modes experienced improvements in learning motivation and self-efficacy, with ChatGPT-assisted learning demonstrating a particularly pronounced capacity to facilitate these outcomes. Previous studies also support this view [45]. A thematic analysis of interviews with higher education experts indicated that the personalized feedback and support provided by ChatGPT can assist students in setting and achieving goals, reflecting on their progress, and enhancing noncognitive skills such as motivation and self-efficacy [46,47].

As an auxiliary tool in medical education, ChatGPT is currently considered a double-edged sword by many scholars [3,48]. On one hand, it may impede the development of students’ critical thinking and independent learning abilities and potentially encourage academic dishonesty [3,49-52]. However, when applied to skill training, ChatGPT displays considerable promise. It is capable of providing tailored assistance and feedback in response to the learner’s progress, offering technical guidance and confirmation of procedural steps [53]. This aids in the comprehension of complex skills, such as tooth preparation, periodontal scaling, and impacted tooth extraction [7]. Furthermore, it diminishes cognitive load during the learning process, enhancing both learning motivation and self-efficacy. Therefore, dental educators must not choke on their knowledge but rather set a new standard for teaching methods and assessment to keep up with the times.

### Limitations

First, it is important to recognize that ChatGPT has some inherent limitation types, although ChatGPT is trained on big data, there is a possibility that the training data may be inherently biased or there may be errors in the training process, which leads to the accuracy and reliability of the information provided will also be affected [54]. During the skill learning process, individuals will have their distinctive queries, so for this study checking the accuracy of the answers to the ChatGPT responses was difficult. Second, this study was conducted using the free version of ChatGPT-3.5, and more research is needed to explore whether ChatGPT-4.0 would be more advantageous in aiding skill instruction [55]. Besides, the study compares ChatGPT with a blank control group based on videos, and further research is required to investigate the detailed strengths and weaknesses of ChatGPT in comparison to other methods.

### Conclusions

This study sought to ascertain whether the supplementary use of ChatGPT-3.5 is more effective for mastering basic oral skills operations among dental students compared with videos alone in terms of manipulative performance, cognitive load, spatial ability, and emotions. The study used desktop VR for skill testing and the results showed that students with the assistance of ChatGPT performed better. Eye-tracking technology was used to record the visual behaviors, and the results revealed that participants in the ChatGPT-3.5 group experienced reduced cognitive load. The interaction analysis highlighted learners with low spatial ability derived greater benefits from the ChatGPT than those with high spatial ability. In addition, the questionnaire revealed that learners in the ChatGPT group demonstrated higher levels of self-efficacy and learning motivation. In conclusion, the findings of this study contribute to recognizing the potential of ChatGPT in dental skills education.

## Appendix

Multimedia Appendix 1 ChatGPT feedback and interactions.

Multimedia Appendix 2 Reference to aSee A6 eye-tracker.

Multimedia Appendix 3 Knowledge test.

Multimedia Appendix 4 CONSORT eHEALTH checklist (V 1.6.1).

## Abbreviations

AI: artificial intelligence
CONSORT-EHEALTH: Consolidated Standards of Reporting Trials of Electronic and Mobile Health Applications and Online Telehealth
VR: virtual reality

## Data Availability

The datasets generated or analyzed during the study are available from the corresponding author on reasonable request.

## References

[1] Gan W, Ouyang J, Li H, Xue Z, Zhang Y, Dong Q, Huang J, Zheng X, Zhang Y (2024). Integrating ChatGPT in orthopedic education for medical undergraduates: randomized controlled trial. J Med Internet Res.

[2] Bagde H, Dhopte A, Alam MK, Basri R (2023). A systematic review and meta-analysis on ChatGPT and its utilization in medical and dental research. Heliyon.

[3] Choi EPH, Lee JJ, Ho M, Kwok JYY, Lok KYW (2023). Chatting or cheating? The impacts of ChatGPT and other artificial intelligence language models on nurse education. Nurse Educ Today.

[4] Lubowitz JH (2023). ChatGPT, an artificial intelligence chatbot, is impacting medical literature. Arthroscopy.

[5] Kitamura FC (2023). ChatGPT is shaping the future of medical writing but still requires human judgment. Radiology.

[6] Lebhar MS, Velazquez A, Goza S, Hoppe IC (2024). Dr. ChatGPT: Utilizing artificial intelligence in surgical education. Cleft Palate Craniofac J.

[7] Karobari MI, Suryawanshi H, Patil SR (2024). Revolutionizing oral and maxillofacial surgery: ChatGPT's impact on decision support, patient communication, and continuing education. Int J Surg.

[8] Arigbede A, Denloye O, Dosumu O (2015). Use of simulators in operative dental education: experience in southern Nigeria. Afr Health Sci.

[9] Dong H, Guo C, Zhou L, Zhao J, Wu X, Zhang X, Zhang X (2022). Effectiveness of case-based learning in Chinese dental education: a systematic review and meta-analysis. BMJ Open.

[10] Li Y, Ye H, Ye F, Liu Y, Lv L, Zhang P, Zhang X, Zhou Y (2021). The current situation and future prospects of simulators in dental education. J Med Internet Res.

[11] Reymus M, Fotiadou C, Kessler A, Heck K, Hickel R, Diegritz C (2019). 3D printed replicas for endodontic education. Int Endod J.

[12] Zeng Y, Ji X, Dong B, Zhang L, Zheng Q, Wang Y, Han X, Ye L, Huang D, Wang S (2024). 3D-printed coloured tooth model for inlay preparation in pre-clinical dental education. Eur J Dent Educ.

[13] Corrêa CG, de Andrade Moreira Machado MA, Ranzini E, Tori R, de Lourdes Santos Nunes F (2017). Virtual reality simulator for dental anesthesia training in the inferior alveolar nerve block. J Appl Oral Sci.

[14] Bruno RR, Wolff G, Wernly B, Masyuk M, Piayda K, Leaver S, Erkens R, Oehler D, Afzal S, Heidari H, Kelm M, Jung C (2022). Virtual and augmented reality in critical care medicine: the patient's, clinician's, and researcher's perspective. Crit Care.

[15] Dai Z, Wang F, Shen C, Ji Y, Li Z, Wang Y, Pu Q (2025). Accuracy of large language models for literature screening in thoracic surgery: Diagnostic study. J Med Internet Res.

[16] Borg A, Georg C, Jobs B, Huss V, Waldenlind K, Ruiz M, Edelbring S, Skantze G, Parodis I (2025). Virtual patient simulations using social robotics combined with large language models for clinical reasoning training in medical education: mixed methods study. J Med Internet Res.

[17] Koo S, Kim A, Donoff RB, Karimbux NY (2015). An initial assessment of haptics in preclinical operative dentistry training. J Investig Clin Dent.

[18] Wajngarten D, Pazos JM, Menegazzo VP, Novo JPD, Garcia PPNS (2021). Magnification effect on fine motor skills of dental students. PLoS One.

[19] Brunken R, Plass JL, Leutner D (2010). Direct measurement of cognitive load in multimedia learning. Educ. Psychol.

[20] Lee E.A.-L., Wong KW (2014). Learning with desktop virtual reality: Low spatial ability learners are more positively affected. Comput Educ.

[21] Makransky G, Lilleholt L (2018). A structural equation modeling investigation of the emotional value of immersive virtual reality in education. Education Tech Research Dev.

[22] Albus P, Vogt A, Seufert T (2021). Signaling in virtual reality influences learning outcome and cognitive load. Comput Educ.

[23] Chen R, Grierson L, Norman G (2015). Manipulation of cognitive load variables and impact on auscultation test performance. Adv Health Sci Educ Theory Pract.

[24] Suebnukarn S, Hataidechadusadee R, Suwannasri N, Suprasert N, Rhienmora P, Haddawy P (2011). Access cavity preparation training using haptic virtual reality and microcomputed tomography tooth models. Int Endod J.

[25] Sweller J, Mestre J, Ross BH (2011). Cognitive Load Theory. Cognition in Education.

[26] Takhdat K, Rebahi H, Rooney DM, Ait Babram M, Benali A, Touzani S, Lamtali S, El Adib AR (2024). The impact of brief mindfulness meditation on anxiety, cognitive load, and teamwork in emergency simulation training: A randomized controlled trial. Nurse Educ Today.

[27] Makransky G, Petersen GB (2019). Investigating the process of learning with desktop virtual reality: A structural equation modeling approach. Comput Educ.

[28] Bandura A (1977). Self-efficacy: toward a unifying theory of behavioral change. Psychol Rev.

[29] Anderman EM, Gray DL, Chang Y (2012). Motivation and classroom learning. Handbook of psychology, Volume 7, Educational psychology. 2nd ed.

[30] From Prohibition to Regulation: Universities Explore the Boundaries of AI Use. MyCOS Research Institute.

[31] Eysenbach G, CONSORT-EHEALTH Group (2011). CONSORT-EHEALTH: improving and standardizing evaluation reports of Web-based and mobile health interventions. J Med Internet Res.

[32] Danesh A, Pazouki H, Danesh F, Danesh A, Vardar-Sengul S (2024). Artificial intelligence in dental education: ChatGPT's performance on the periodontic in-service examination. J Periodontol.

[33] Ali K, Barhom N, Tamimi F, Duggal M (2024). ChatGPT-A double-edged sword for healthcare education? Implications for assessments of dental students. Eur J Dent Educ.

[34] Liu M, Okuhara T, Chang X, Shirabe R, Nishiie Y, Okada H, Kiuchi T (2024). Performance of ChatGPT across different versions in medical licensing examinations worldwide: systematic review and meta-analysis. J Med Internet Res.

[35] Castner N, Appel T, Eder T, Richter J, Scheiter K, Keutel C, Hüttig F, Duchowski A, Kasneci E (2020). Pupil diameter differentiates expertise in dental radiography visual search. PLoS One.

[36] Mooney P, Cui W, Guan B, Juhász L (2023). Towards understanding the geospatial skills of ChatGPT: Taking a geographic information systems (GIS) exam.

[37] Renshaw A, Lourentzou I, Lee J, Crawford T, Kim J (2025). Comparing the spatial querying capacity of large language models: OpenAI's ChatGPT and Google's gemini pro. Prof. Geogr.

[38] Sobo A, Mubarak A, Baimagambetov A, Polatidis N (2024). Evaluating LLMs for code generation in HRI: A comparative study of ChatGPT, Gemini, and Claude. Appl. Artif. Intell.

[39] Sarilita E, Lita YA, Firman DR, Wilkinson T, Susilawati S, Saptarini R, Aripin D, Sjamsudin E (2022). Spatial ability and anatomy learning performance among dental students. Korean J Med Educ.

[40] Collet P, Tra R, Reitmann A, Valette S, Hoyek N, Maurin J, Ducret M, Villat C, Santamaria J, Richert R (2025). Spatial abilities and endodontic access cavity preparation: Implications for dental education. Eur J Dent Educ.

[41] Nilsson T, Hedman L, Ahlqvist J (2007). Visual-spatial ability and interpretation of three-dimensional information in radiographs. Dentomaxillofac Radiol.

[42] Gonzales RA, Ferns G, Vorstenbosch MATM, Smith CF (2020). Does spatial awareness training affect anatomy learning in medical students?. Anat Sci Educ.

[43] Goodacre CJ (2018). Digital learning resources for prosthodontic education: the perspectives of a long-term dental educator regarding 4 key factors. J Prosthodont.

[44] Sweller J, Ayres P, Kalyuga S, Sweller J, Ayres P, Kalyuga S (2011). The Expertise Reversal Effect. Cognitive Load Theory.

[45] Sauder M, Tritsch T, Rajput V, Schwartz G, Shoja MM (2024). Exploring generative artificial intelligence-assisted medical education: assessing case-based learning for medical students. Cureus.

[46] Xu X, Wang X, Zhang Y, Zheng R (2024). Applying ChatGPT to tackle the side effects of personal learning environments from learner and learning perspective: An interview of experts in higher education. PLoS One.

[47] Schiefele U, Krapp A, Winteler A, Renninger KA, Hidi S, Krapp A (1992). Interest as a predictor of academic achievement: A meta-analysis of research. The role of interest in learning and development.

[48] Abd-Alrazaq A, AlSaad R, Alhuwail D, Ahmed A, Healy PM, Latifi S, Aziz S, Damseh R, Alabed Alrazak S, Sheikh J (2023). Large language models in medical education: Opportunities, challenges, and future directions. JMIR Med Educ.

[49] Lee H (2024). The rise of ChatGPT: Exploring its potential in medical education. Anat Sci Educ.

[50] Gödde D, Nöhl S, Wolf C, Rupert Y, Rimkus L, Ehlers J, Breuckmann F, Sellmann T (2023). A SWOT (strengths, weaknesses, opportunities, and threats) analysis of ChatGPT in the medical literature: Concise review. J Med Internet Res.

[51] Arif TB, Munaf U, Ul-Haque I (2023). The future of medical education and research: Is ChatGPT a blessing or blight in disguise?. Med Educ Online.

[52] Safranek CW, Sidamon-Eristoff AE, Gilson A, Chartash D (2023). The role of large language models in medical education: Applications and implications. JMIR Med Educ.

[53] Wang Y, Chen Y, Sheng J (2024). Assessing ChatGPT as a medical consultation assistant for chronic hepatitis B: Cross-language study of English and Chinese. JMIR Med Inform.

[54] Shimizu I, Kasai H, Shikino K, Araki N, Takahashi Z, Onodera M, Kimura Y, Tsukamoto T, Yamauchi K, Asahina M, Ito S, Kawakami E (2023). Developing medical education curriculum reform strategies to address the impact of generative AI: Qualitative study. JMIR Med Educ.

[55] Takagi S, Watari T, Erabi A, Sakaguchi K (2023). Performance of GPT-3.5 and GPT-4 on the Japanese medical licensing examination: comparison study. JMIR Med Educ.

