# Supplementary material for: Exploring the Application Capability of ChatGPT as an Instructor in Skills Education for Dental Medical Students: Randomized Controlled Trial
Source: J Med Internet Res. 2025 May 27;27:e68538. doi: 10.2196/68538 (PMC12152432; doi:10.2196/68538)
Supplement: Multimedia Appendix 1 [file jmir_v27i1e68538_app1.docx]

ChatGPT feedback and interactions

| **Questions** | **Answers** |
| --- | --- |
| **When utilizing Gracey curettes for subgingival debridement and root planning, how to differentiate the working end and cutting edges?** | Accurate identification and utilization of the working end and working blade of the Gracey curette, as well as selecting the appropriate working head for each specific tooth surface, are crucial during subgingival scaling and root planning. The following is a detailed guide:   1. **Identification of the Working End and Working Blade:**  - **Determination of the Working End:**   - The working end of the Gracey curette is designed with a single-sided cutting edge, tailored for specific tooth surfaces and areas.   - Typically, the working end is designed to form a 70° angle with the tooth surface to ensure effective scaling.   - When placing the working end of the curette on the mesial or distal surface of the target tooth, ensure that the working blade is in close contact with the tooth surface. At this time, the neck of the curette should be roughly parallel to the long axis of the tooth. - **Identification of the Working Blade:**   - Hold the curette vertically with the tip of the working end facing yourself and the short handle perpendicular to the ground.   - The lower cutting edge at this position is the working blade.   - The working blade is usually on the side away from the short handle, while the non-working blade is closer to the short handle.  1. **Selection of Appropriate Models for Specific Tooth Surfaces:**   Gracey curettes come in various models, each designed for specific teeth and surfaces.   - **Anterior Teeth:**   - #1/2, #3/4: Suitable for all surfaces of anterior teeth.   - #5/6: Suitable for all surfaces of anterior teeth and canines. - **Posterior Teeth:**   - #7/8, #9/10: Suitable for the buccal and lingual surfaces of posterior teeth.   - #11/12: Suitable for the mesial surfaces of posterior teeth.   - #13/14: Suitable for the distal surfaces of posterior teeth.   - #15/16: Suitable for the mesial surfaces of posterior teeth, similar in design to #11/12 but with adjusted angles to better adapt to the posterior region.   - #17/18: Suitable for the distal surfaces of posterior teeth, similar in design to #13/14 but with adjusted angles to better adapt to the posterior region.   **3. Steps for Selecting the Curette:**   - Determine the Location and Surface of the Tooth to Be Treated: First, clearly identify the specific tooth and surface (mesial, distal, buccal, or lingual) that requires scaling. - Select the Appropriate Model of Gracey Curette: Choose the model that corresponds to the tooth position and surface according to the classification above. - Verify the Fit of the Working End: Place the working end of the selected curette on the tooth surface to ensure it fits well and that the working blade is oriented towards the tooth surface. - Adjust the Holding Angle: When holding the curette, ensure that the handle forms an appropriate angle with the long axis of the tooth to allow even force application and ensure effective scaling.   By accurately identifying the working end and working blade of the Gracey curette and selecting the appropriate working head for each specific tooth surface, the efficacy of subgingival scaling and root planning can be enhanced, ensuring precise treatment and patient comfort. |
| **In periodontal subgingival scaling and root planning, how to choose the appropriate finger rest, operator’s position, patient’s chair position, and light direction according to different tooth locations?** | During periodontal subgingival scaling and root planning, it is crucial to adjust the operator’s position, patient’s chair and head position, finger rest, and light position according to different tooth areas (upper and lower jaws, left and right sides, anterior and posterior teeth) and the orientation of the tooth surface (surfaces facing away from or towards the operator). This ensures the precision and comfort of the procedure. The detailed guidance is as follows:  **1. Operator’s Position:**   - **Maxillary Anterior Teeth:**   - Labial Surface: The operator should be positioned directly in front of the patient (12 o'clock position) for direct vision.   - Lingual Surface: The operator should be positioned directly in front of the patient (12 o'clock position) and use a mouth mirror for indirect vision. - **Mandibular Anterior Teeth:**   - Labial Surface: The operator should be positioned at the patient’s right front side (7–8 o'clock position) for direct vision.   - Lingual Surface: The operator should be positioned at the patient’s right front side (7–8 o'clock position) and use a mouth mirror for indirect vision. - **Maxillary Right Posterior Teeth:**   - Buccal Surface: The operator should be positioned on the patient’s right side (9 o'clock position) for direct vision.   - Palatal Surface: The operator should be positioned on the patient’s right side (9 o'clock position) and use a mouth mirror for indirect vision. - **Maxillary Left Posterior Teeth:**   - Buccal Surface: The operator should be positioned on the patient’s left side (3 o'clock position) for direct vision.   - Palatal Surface: The operator should be positioned on the patient’s left side (3 o'clock position) and use a mouth mirror for indirect vision. - **Mandibular Right Posterior Teeth:**   - Buccal Surface: The operator should be positioned on the patient’s right side (9 o'clock position) for direct vision.   - Lingual Surface: The operator should be positioned on the patient’s right side (9 o'clock position) and use a mouth mirror for indirect vision. - **Mandibular Left Posterior Teeth:**   - Buccal Surface: The operator should be positioned on the patient’s left side (3 o'clock position) for direct vision.   - Lingual Surface: The operator should be positioned on the patient’s left side (3 o'clock position) and use a mouth mirror for indirect vision.   **2. Patient’s Chair and Head Position:**   - **Maxillary Teeth:**   - Chair Position: The patient should be in a supine position with the chair back nearly parallel to the floor.   - Head Position: The head should be slightly tilted backward, with the maxillary occlusal plane forming a 45°–90° angle with the floor. - **Mandibular Teeth:**   - Chair Position: The patient should be in a supine position with the chair back forming a 15°–20° angle with the floor.   - Head Position: The head should be slightly tilted forward, with the mandibular occlusal plane as parallel to the floor as possible.   **3. Finger Rest:**   - **Principle of Selection:** An intraoral rest should be used, typically with the middle finger or the middle and ring fingers together. The rest should be as close as possible to the tooth being treated and should move with changes in the working area. - **Specific Operations:**   - Anterior Teeth: Establish a rest on the adjacent tooth surface to ensure stability.   - Posterior Teeth: Establish a rest on the same-side or contralateral tooth surface. An opposing arch rest or extraoral rest may be used if necessary.   **4. Light Position:**   - **Maxillary Teeth:** The surgical light should be positioned to shine from the direction of the patient’s chest, with the light beam perpendicular to the maxillary teeth to ensure a clear field of vision. - **Mandibular Teeth:** The surgical light should be positioned to shine from the direction of the patient’s head, with the light beam angled to illuminate the mandibular teeth adequately.   By adjusting the operator’s position, patient’s chair and head position, finger rest, and light position according to different tooth areas and surface orientations, the efficiency and effectiveness of subgingival scaling and root planning can be enhanced. This also ensures patient comfort and the success of the treatment. |
| **In the endodontic access procedure of the maxillary first molar, where should I start, and how should I control the direction, depth, and extent of the bur penetration to prevent perforation of the pulp chamber walls and floor?** | When performing access cavity preparation on the maxillary first molar, precise positioning and control of the bur’s direction, depth, and operative range are crucial to avoid lateral and apical perforations. Below is a detailed guide:  **1. Selection of the Entry Point:**   - **Location:** The entry point for access should be located in the central fossa of the occlusal surface, slightly towards the mesiobuccal cusp, typically between the mesiobuccal and distobuccal cusps. - **Rationale:** Choosing this point facilitates direct access to the pulp chamber, minimizes damage to tooth structure, and avoids deviation from the chamber’s center.   **2. Bur Selection and Initial Penetration:**   - **Bur Choice:** Use a high-speed round bur (e.g., #4 round bur) for initial penetration. - **Penetration Technique:** Begin perpendicular to the occlusal surface at the selected entry point, gradually advancing into the dentin layer. Maintain the bur parallel to the long axis of the tooth to ensure accurate entry into the pulp chamber.   **3. Control of Bur Direction and Depth:**   - **Direction Control:**   - During initial penetration, the bur should be perpendicular to the occlusal surface.   - After entering the dentin layer, gradually adjust the bur to align with the long axis of the tooth. This adjustment helps avoid deviation from the center of the pulp chamber and prevents lateral perforation. - **Depth Control:**   - Pay attention to tactile feedback during penetration. A “drop-in” sensation indicates entry into the pulp chamber.   - Upon feeling this sensation, cease further downward cutting to avoid over-penetration and apical perforation.   **4. Enlargement of the Pulp Chamber Access:**   - **Technique:** After entering the pulp chamber, use the side of the round bur in a pulling motion to remove the chamber roof and enlarge the access opening. - **Precautions:** Avoid excessive removal of tooth structure to prevent damage to the chamber floor or walls.   **5. Location and Opening of the Canal Orifices:**   - **Instrumentation:** Use an endodontic explorer (e.g., DG16 double-ended explorer) to locate the canal orifices within the pulp chamber. - **Procedure:** Once the orifices are identified, use appropriate instruments to open them, ensuring smooth progression of subsequent procedures.   **Key Considerations:**   - **Visibility and Illumination:** Maintain clear visibility and adequate lighting throughout the procedure to ensure precision. - **Irrigation:** Regularly irrigate the pulp chamber with saline or sodium hypochlorite solution to remove debris and prevent infection. - **Operative Force:** Use gentle and controlled movements to avoid excessive force, which may damage tooth structure.   **Conclusion:**  By meticulously following these steps, lateral and apical perforations can be effectively avoided, ensuring successful access cavity preparation on the maxillary first molar. Each step requires careful attention and patience to protect tooth structure and facilitate successful endodontic treatment. |
| **Fabrication Steps for Primary Tooth Preformed Crowns.** | **1.Preoperative Evaluation and Preparation**   - **Indication Assessment**: SSCs are indicated for primary teeth with extensive caries, post-endodontic treatment, enamel hypoplasia, or structural defects. - **Tooth Evaluation**: Ensure the abutment tooth is healthy without severe caries or periodontal involvement. - **Material Preparation**: Gather SSC kits (crown scissors, contouring pliers, metal finishing burs) and auxiliary materials (e.g., rubber dam).   **2. Tooth Preparation**   - **Tooth Cleaning**: Remove carious tissue and clean the tooth surface thoroughly. - **Proximal Preparation**:   - Prepare mesial and distal surfaces to be parallel or slightly convergent.   - For the distal surface of the second primary molar (if it is the last tooth in the arch), extend the preparation slightly subgingivally. - **Buccal/Lingual Preparation**:   - Generally unnecessary unless prominent buccal cervical contours exist. If present, reduce the buccal cervical bulge to prevent excessive crown-tooth gaps. - **Occlusal Reduction**:   - Remove approximately 1.0 mm of occlusal tissue, paying special attention to reducing occlusal ridges for proper crown adaptation.   **3. Crown Selection and Trial Fitting**   - **Size Selection**: Choose an SSC based on the mesiodistal width or crown circumference of the prepared tooth. - **Trial Placement**: Test the crown’s fit, ensuring passive seating without excessive tightness or looseness.   **4.Crown Adjustment**   - **Height and Margin Trimming**: Trim the crown height and contour the cervical margin to achieve a subgingival extension of 0.5–1.0 mm. - **Anatomical Shaping**: Use crown pliers to adjust occlusal contours, restore physiological convexity, and tighten the cervical margin to mimic natural morphology.   **5. Margin Polishing and Final Fitting**   - **Polishing**: Smooth trimmed margins with fine-grit discs or rubber wheels to prevent gingival irritation. - **Final Check**: Verify proper occlusion, cervical adaptation, axial alignment, and harmony with adjacent teeth.   **6. Cementation**   - **Cement Selection**: Use glass ionomer cement or polycarboxylate zinc cement. - **Cementation Protocol**: Apply cement to the crown’s inner surface, seat the crown with firm pressure, and remove excess material after initial set.   **7. Postoperative Care and Maintenance**   - **Patient Instructions**:   - Mild discomfort or occlusal sensitivity may occur on the day of placement; symptoms typically resolve within a week.   - Avoid chewing on the treated tooth for 2 hours and hard foods for 24 hours.   - Refrain from sticky foods (e.g., caramel, nougat). If the crown dislodges, seek immediate re-cementation. - **Oral Hygiene**: Educate caregivers/children on proper brushing to prevent caries and periodontal issues. - **Follow-Up**: Schedule reviews every 3–6 months to assess crown retention, abutment tooth health, and permanent tooth eruption. |
| **Gingivoplasty procedures for Gingival Hyperplasia.** | Gingivoplasty is a surgical procedure to reshape gingival tissue, improving both aesthetics and function, commonly used to treat gingival hyperplasia (e.g., drug-induced, chronic inflammatory hyperplasia). Below are the detailed surgical steps and key considerations:  **I.Preoperative Preparation**   1. **Diagnosis and Indication Assessment**    - Identify the cause of hyperplasia (e.g., drug-induced, inflammatory, genetic) and rule out systemic conditions (e.g., leukemia, coagulation disorders).    - Imaging: Take periapical radiographs or CBCT to evaluate alveolar bone morphology and periodontal support.    - For drug-induced hyperplasia (e.g., due to calcium channel blockers or antiepileptics), consult with the prescribing physician to adjust medications if possible. 2. **Patient Preparation**    - **Oral Hygiene**: Perform full-mouth scaling (supragingival and subgingival) to control periodontal inflammation.    - **Informed Consent**: Discuss risks, benefits, and postoperative care.    - **Anesthesia**: Administer local anesthesia (e.g., 2% lidocaine with epinephrine) via infiltration or nerve block.   **II. Surgical Procedure**  **1.Marking the Gingival Contour**   - Use a periodontal probe or marking pen to outline the desired physiological gingival margin (scalloped contour) as a guide for excision. - Key: Preserve at least 2 mm of keratinized gingiva to avoid postoperative recession.   **2. Excision of Hyperplastic Tissue**   - **Instrumentation**:   - Scalpel (No. 15 or 12 blade).   - Electrosurgical unit (for hemostasis or fine adjustments).   - Laser (Er:YAG or CO₂ lasers to minimize bleeding and promote healing). - **Incision Design**:   - **External Bevel Incision**: Position the blade at a 45° angle to the tooth’s long axis, excising excess tissue from the marked line toward the root.   - **Depth**: Remove hyperplastic tissue until the normal sulcus is exposed, avoiding damage to alveolar bone.  1. **Contouring the Gingiva**  - Refine the gingival margin with a high-speed round bur or diamond stone to create a smooth scalloped contour. - **Interdental Papilla**: Recreate natural triangular shapes to prevent “black triangles.”   - Hemostasis and Wound Management - **Pressure Hemostasis**: Apply saline-soaked gauze. - **Electrocautery**: Address active bleeding points. - **Periodontal Dressing**: Apply Coe-Pak to protect the wound and reduce sensitivity.   **4. Immediate Postoperative Care**   - Ice application (20 minutes) to minimize swelling. - Prescribe analgesics (e.g., ibuprofen) and antibiotics (e.g., amoxicillin) if needed.   **III. Postoperative Care and Follow-Up**   1. **Patient Instructions**    - Avoid brushing/flossing for 24 hours; use 0.12% chlorhexidine mouthwash twice daily.    - Maintain a soft diet for 1 week; avoid hot/spicy foods.    - Schedule a 7-day follow-up to remove the dressing and assess healing. 2. **Long-Term Management**    - **Oral Hygiene**: Teach Bass technique and interdental cleaning tools.    - **Periodontal Maintenance**: Review every 3–6 months to prevent recurrence.    - **Drug-Induced Cases**: Monitor and collaborate with physicians for medication adjustments.   **IV. Complications and Management**   1. **Postoperative Bleeding**: Apply pressure, sutures, or hemostatic agents. 2. **Infection**: Enhance oral hygiene and prescribe antibiotics. 3. **Suboptimal Contour**: Perform secondary refinement if necessary. 4. **Recurrence**: Address underlying causes (e.g., medication changes, inflammation control).   **V. Key Technical Tips**   - **Precise Marking**: Base contour design on healthy gingival anatomy. - **Incremental Tissue Removal**: Avoid over-reduction to preserve keratinized tissue. - **Balance Function and Aesthetics**: Align the gingival margin with the dental arch curvature; avoid over-thinning interdental areas.   By adhering to standardized protocols and postoperative care, gingivoplasty effectively restores gingival form and periodontal health. For complex cases, use magnification (e.g., microscope) to enhance precision. |
| **How to extract a mesioangularly impacted mandibular third molar?** | The removal of a mesioangularly impacted mandibular third molar is a complex procedure requiring meticulous preoperative evaluation and precise execution. Below are the critical steps and precautions:  **1. Preoperative Assessment**   - **Imaging**:   - Obtain a panoramic radiograph or CBCT (cone beam computed tomography) to evaluate:     - Tooth position and angulation.     - Relationship to adjacent structures (e.g., inferior alveolar nerve canal).     - Root morphology (e.g., curvature, fusion). - **Medical History**:   - Assess systemic health (e.g., bleeding disorders, diabetes) and rule out contraindications.   **2. Anesthesia**   - **Inferior Alveolar Nerve Block**:   - Administer local anesthesia (e.g., 2% lidocaine with 1:100,000 epinephrine).   - Supplement with buccal infiltration if needed for complete analgesia.   **3. Incision Design**   - **Location**:   - Begin at the distobuccal gingival sulcus of the second molar, extending posteriorly along the gingival margin to the distal aspect of the impacted tooth. If necessary, extend into the retromolar area. - **Type**:   - Use a linear incision or envelope flap with a releasing incision to ensure adequate access.   **4. Flap Elevation**   - **Technique**:   - Elevate a mucoperiosteal flap using a periosteal elevator to expose the impacted tooth and surrounding bone. - **Precautions**:   - Avoid trauma to adjacent soft tissues and neurovascular bundles.   **5. Bone Removal**   - **Objective**:   - Remove overlying bone to expose the crown and partial root structure. - **Method**:   - Use a **high-speed handpiece with round or fissure burs** to remove bone incrementally.   - Preserve the inferior alveolar nerve by maintaining a safe distance (≥2 mm).   **6. Tooth Sectioning**   - **Strategy**:   - For mesioangular impactions with a large crown:     - **Section the crown** into mesial and distal portions.     - **Remove the distal segment first** by creating a buccolingual groove, splitting it into halves for extraction.     - **Displace the mesial segment** into the distal space, then section and remove it. - **Tools**:   - High-speed handpiece with tapered fissure burs.   **7. Root Extraction**   - **Technique**:   - Use elevators (e.g., straight or Cryer) or forceps to luxate and deliver the root along its long axis. - **Precautions**:   - Apply controlled force to avoid root fracture or nerve injury.   **8. Wound Management**   - **Debridement**:   - Irrigate the socket to remove debris (bone/tooth fragments, granulation tissue). - **Suturing**:   - Close the flap with 3-0 or 4-0 absorbable sutures if necessary.   **9. Postoperative Care**   - **Hemostasis**:   - Bite on a sterile gauze pad for **30–40 minutes**. - **Diet**:   - Avoid eating for 2 hours; then transition to soft, cool foods. - **Oral Hygiene**:   - No rinsing or brushing for 24 hours. Resume gently with chlorhexidine mouthwash afterward. - **Follow-Up**:   - Schedule a review to monitor healing and address complications.   **Key Precautions**   - **Preoperative Communication**:   - Discuss risks (e.g., nerve injury, dry socket) and obtain informed consent. - **Intraoperative Technique**:   - Prioritize gentle handling, minimal bone removal, and nerve protection. - **Postoperative Monitoring**:   - Observe the patient for 30 minutes post-surgery to detect early complications.   Successful extraction of mesioangularly impacted mandibular third molars demands expertise in surgical planning, precise instrumentation, and adherence to anatomical safeguards. Mastery of these principles minimizes complications and ensures optimal outcomes. |
| **Tooth Preparation for a Metal-Ceramic Full Crown on the Mandibular First Molar.** | The preparation of the abutment tooth for a metal-ceramic crown aims to provide adequate space for the restoration, ensure retention, and achieve optimal functional and aesthetic outcomes. Below are the detailed preparation steps:   - **Occlusal Surface Preparation** - **Depth Orientation Grooves**:   - Use a cylindrical diamond bur or dedicated depth-cutting bur to create 1.5 mm deep grooves on functional and non-functional cusps of the occlusal surface. - **Uniform Reduction**:   - Follow the anatomical contours of the occlusal surface, using the grooves as a guide, to evenly remove tooth structure. Maintain natural occlusal morphology to ensure sufficient clearance in centric occlusion, protrusion, and lateral excursions. - **Buccolingual Surface Preparation** - **Staged Preparation**:   - Divide the buccal and lingual surfaces into **occlusal half** and **gingival half** due to their natural curvature. - **Guided Reduction**:   - Create depth grooves in the occlusal half with a cylindrical diamond bur to eliminate undercuts between the crown margin and the height of contour. Align axial walls with the path of insertion. - **Anatomical Contouring**:   - From the height of contour to the occlusal edge, follow the tooth’s natural shape to achieve uniform reduction, preserving the original buccolingual contours. - **Proximal Surface Preparation** - **Stealth Grinding Technique**:   - Use a fine tapered diamond bur to perform intermittent grinding along the proximal surfaces (mesial and distal) in a buccolingual direction. Maintain a stable fulcrum and adjust the angulation to gradually open the contact area while preserving adequate space. - **Convergence Control**:   - Ensure the proximal walls are parallel or slightly convergent (6–10° taper) to the path of insertion. - **Cervical Margin Preparation** - **Shoulder Design**:   - Choose a 90° rounded shoulder or 135° shallow chamfer based on retention needs, crown height, and gingival position.   - **Margin Placement**:     - Subgingival (0.5 mm below gingival crest), equigingival, or supragingival (for posterior teeth). - **Margin Refinement**:   - Ensure a continuous, smooth, and non-beveled finish without sharp edges. - **Axial Line Angle Preparation** - **Line Angle Blending**:   - Round all line angles (e.g., bucco-occlusal, linguo-occlusal) to eliminate sharp transitions and ensure uniform reduction. - **Root Furcation Area**:   - Prepare adequate clearance near the root bifurcation to harmonize the crown margin with the furcation anatomy. - **Finishing and Polishing** - **Smooth Transitions**:   - Refine all surfaces with a fine-grit diamond bur or tungsten carbide polishing bur to eliminate rough areas and sharp edges. - **Final Inspection**:   - Verify smoothness, adequate reduction (1.5–2 mm occlusally, 1.0–1.5 mm axially), and proper margin continuity.   **Key Considerations**   - **Preservation of Tooth Structure**: Avoid over-reduction to maintain pulp vitality and structural integrity. - **Biomechanical Harmony**: Ensure even stress distribution and passive fit of the final restoration. - **Gingival Health**: Minimize trauma to soft tissues during margin preparation.   By following these steps, clinicians can create an ideal foundation for a metal-ceramic crown, ensuring durability, aesthetics, and patient comfort. |
| **How to perform tooth preparation for a proximal-occlusal inlay?** | **I.Preoperative Evaluation and Design**  **1.Indication Assessment**   - Indications include moderate to severe caries involving the proximal and occlusal surfaces (without pulp exposure), tooth fracture, or replacement of old restorations. - Contraindications: pulp exposure, severe periodontal disease, or insufficient remaining tooth structure to provide retention.   **2.Radiographic Examination**   - Use periapical or bitewing radiographs to assess the depth of caries, the extent of the proximal contact area, and the relationship with adjacent teeth, and to rule out periapical lesions.   **II. Tooth Preparation Steps**   - **Removal of Carious Tissue** - **Instrument Selection**:   - High-speed round bur (for removing large amounts of decay) → Low-speed round bur or excavator (for fine cleaning of the near-pulp area). - **Precautions**:   - When soft dentin is retained near the pulp, an indirect pulp capping agent (e.g., calcium hydroxide) should be used. - **Proximal Cavity Preparation** - **Proximal Extension**:   - Use a fine conical diamond bur (e.g., TF-13) to extend from the occlusal marginal ridge to the proximal surface, forming a **b**ox-shaped cavity.   - **Key Parameters**:     - **Proximal cavity wall**: Parallel to the long axis of the tooth or flared 2–5°, with a height ≥1.5 mm.     - **Gingival wall**: Located 0.5–1.0 mm above the gingiva, with a width ≥1.0 mm and a gap ≥0.5 mm from the adjacent tooth to prevent adhesion. - **Edge Treatment**:   - Prepare a **shallow concave edge (0.3–0.5 mm width)** on the gingival wall of the proximal surface to enhance marginal seal. - **Occlusal Cavity Preparation** - **Dovetail Retention Design**:   - Use a conical diamond bur (e.g., 856-014) to prepare the dovetail structure:     - **Dovetail isthmus**: Located in the central fossa, with a width of 1/3–1/2 of the buccolingual diameter and a depth of 1.5–2.0 mm.     - **Dovetail expansion**: Extend towards the distal surface, avoiding the cusp oblique plane, and connect continuously with the proximal box-shaped cavity. - **Occlusal Bevel Preparation**:   - Prepare a **45° short bevel (approximately 0.5 mm wide)** on the cavity margin to reduce stress concentration and improve marginal fit. - **Finishing and Polishing** - **Smooth Cavity Walls**:   - Use a **tungsten carbide polishing bur** to smooth the cavity walls, eliminate undercuts, and ensure rounded line angles (avoid sharp angles). - **Edge Enhancement**:   - Polish the proximal gingival edge with a **flame-shaped bur** to form a smooth, continuous concave termination line.   **III. Key Details and Precautions**   1. **Proximal Protection**:    - Place a **metal matrix band** or **transparent matrix system** to isolate the adjacent tooth and prevent accidental damage.    - Use a **gingival retractor** to expose the gingival wall and ensure a clear view. 2. **Resistance Form Design**:    - Avoid excessive extension to the cusp oblique plane; the dovetail expansion should avoid the functional contact area of the cusp.    - The remaining tooth thickness should be ≥2.0 mm (to prevent fracture). 3. **Path of Insertion Control**:    - All cavity walls (proximal and occlusal) should be **parallel to the path of insertion** to prevent interference during the seating of the inlay. 4. **Temporary Restoration**:    - Use **flowable resin** to temporarily seal and protect the pulp, maintaining proximal contact.   **IV. Common Errors and Avoidance Strategies**   - **Error 1: Excessive inward inclination of the proximal cavity wall** → Leads to difficulty in seating the inlay. **Solution**: Use a parallelometer to assist in controlling the direction of the bur. - **Error 2: Gingival wall positioned too deep** → Stimulates the gingiva or damages the biological width. **Solution**: Measure the gingival sulcus depth with a probe before the procedure; the gingival wall should be at least 0.5 mm above the gingiva. - **Error 3: Insufficient occlusal preparation** → The inlay may be too thin and prone to fracture. **Solution**: Use the depth-guiding groove method (initial groove depth of 1.5 mm, gradually deepened to 2.0 mm).   **V. Postoperative Management and Restoration Selection**   - **Impression Making**: Use a two-step polyvinyl siloxane impression to ensure clear details of the proximal and gingival margins. - **Material Selection**:   - **High-strength resin inlay for posterior teeth**: Suitable for areas with moderate occlusal forces.   - **Lithium disilicate glass ceramic inlay**: Suitable for cases requiring both aesthetics and strength.   By accurately preparing the tooth structure and paying attention to details, proximal-occlusal inlays can effectively restore the shape and function of the tooth while achieving a minimally invasive treatment goal. |
| **How to arrange teeth in complete dentures?** | The arrangement of teeth in complete dentures is a crucial step in restoring edentulous patients, balancing function, aesthetics, and stability. Below is a scientific and standardized process for tooth arrangement, along with key points:  **I. Preparations Before Tooth Arrangement**  **1. Determination of Occlusal Relationship**   - **Vertical Dimension**:   - Determine the vertical height using the rest position method (2–4 mm gap) or the phonetic method (minimum gap during the pronunciation of the “s” sound). - **Horizontal Relationship**:   - Record the centric relation (CR) to ensure the condyles are centrally positioned in the glenoid fossa. - **Selection of Artificial Teeth** - **Anterior Teeth**:   - Choose the tooth type (square, ovoid, or tapered) based on the patient’s facial morphology (face shape and lip protrusion). - **Posterior Teeth**:   - Select anatomical teeth (for high alveolar ridge resorption) or semi-anatomical teeth (for low resorption) based on the degree of alveolar ridge resorption. - **Marking Reference Lines** - **Midline**:   - Align with the nasal root, philtrum, and chin midline. - **Commissure Line**:   - Determine the width of anterior teeth (the distance between the commissures is approximately equal to the total width of six anterior teeth). - **Smile Line**:   - The incisal edge of maxillary anterior teeth should be consistent with the arc of the lower lip during smiling.   **II. Principles of Anterior Tooth Arrangement**   - **Maxillary Anterior Teeth** - **Vertical Position**:   - The incisal edge should be 2 mm below the upper lip (for young patients) or level with it (for elderly patients). - **Labiolingual Position**:   - Slightly inward at the cervical area, with the incisal edge slightly labially inclined to create a natural prominence. - **Mesiodistal Position**:   - Align with the midline, with lateral incisors slightly distal and canines positioned inside the commissure line. - **Mandibular Anterior Teeth** - **Overjet and Overbite**:   - Overjet of 1–2 mm and overbite of 1 mm (to avoid deep overbite leading to anterior dislocation of the denture). - **Arrangement Curve**:   - Parallel to the crest of the mandibular alveolar ridge, avoiding excessive lingual inclination (which may affect tongue movement).   **III. Methods for Posterior Tooth Arrangement**   - **Neutral Zone Principle** - **Lingual Boundary**:   - The lingual cusp of posterior teeth should be 1–2 mm lingual to the crest of the alveolar ridge to avoid interference with the tongue. - **Buccal Boundary**:   - Maintain a 1 mm gap between the buccal surface of the teeth and the inner cheek mucosa. - **Occlusal Plane Positioning** - **Anterior Portion**:   - Parallel to the interpupillary line. - **Posterior Portion**:   - Parallel to the nasolabial-auricular line, with the lingual side slightly lower than the buccal side (to compensate for tongue movement). - **Balanced Occlusion Design** - **Anatomical Teeth**:   - Bilateral balanced occlusion (with three-point contact during protrusive and lateral movements). - **Semi-Anatomical Teeth**:   - Lingualized occlusion (reducing lateral forces, suitable for patients with severe alveolar ridge resorption).   **IV. Occlusal Adjustment and Verification**   - **Protrusive Balance** - Light contact of anterior teeth, with at least one pair of posterior teeth (usually the second molars) maintaining contact. - **Lateral Balance** - On the working side (chewing side), the buccal cusp of posterior teeth should contact, while the lingual cusp contacts on the balancing side. - **Occlusal Paper Check** - Use 20 μm occlusal paper to eliminate early contact points and ensure even contact.   **V. Trial Placement and Aesthetic Fine-Tuning**  **1. Facial Harmony Assessment**   - **Front View**:   - The exposure of anterior teeth and smile line should match the patient’s age. - **Side View**:   - Lip support (to avoid the “denture face” appearance). - **Patient Feedback Adjustment** - **Phonetic Testing**:   - During the pronunciation of “f” and “v” sounds, the incisal edge of the maxillary teeth should lightly touch the inner side of the lower lip. - **Comfort**:   - Modify teeth that excessively press on the gums or lingual side.   **VI. Management of Special Cases**   - **Severe Alveolar Ridge Resorption** - Posterior teeth should be arranged more lingually, reducing the buccolingual width (to decrease lateral forces). - Use a shortened dental arch design (reducing to the first molar). - **High Smile Line Patients** - The cervical exposure of maxillary anterior teeth should be coordinated with the gingival contour, and pink resin can be used to modify the gingival margin if necessary.   **Key Points to Note**   - **Function First**:   - Occlusal stability > Aesthetics; avoid excessive protrusion of anterior teeth leading to denture dislocation. - **Tongue Space Preservation**:   - Maintain a functional space of 3–5 mm between the lingual side of posterior teeth and the tongue. - **Mucoperiosteal Protection**:   - For patients with flat alveolar ridges, avoid sharp cusps (to reduce localized pressure).   Through systematic tooth arrangement and meticulous adjustment, complete dentures can achieve good masticatory efficiency, phonetic function, and natural appearance, significantly improving the quality of life for edentulous patients. |
| **Inferior Alveolar Nerve Block: A Step-by-Step Guide.** | The inferior alveolar nerve block is one of the most commonly used anesthetic techniques in oral and maxillofacial surgery. It is suitable for diagnostic and therapeutic procedures involving the teeth, periodontal tissues, and soft tissues on the same side of the mandible, such as tooth extraction, root canal treatment, and alveolar surgery. The core objective is to precisely anesthetize the inferior alveolar nerve while avoiding damage to adjacent blood vessels and nerves. Below are the standardized steps and precautions for performing this procedure:  **I. Anatomical Localization**  **Target Nerve**: The inferior alveolar nerve (a branch of the mandibular division of the trigeminal nerve) enters the mandibular canal through the mandibular foramen and innervates the ipsilateral mandibular teeth, alveolar bone, and buccal gingiva up to the midline.  **Key Landmarks**:   1. **Pterygomandibular Fold**: A longitudinal mucosal fold on the inner side of the buccal mucosa. 2. **Retromolar Pad**: The soft tissue elevation distal to the last mandibular molar. 3. **Lingula**: A bony projection on the medial side of the mandibular ramus, marking the location of the mandibular foramen.   **II. Procedure Steps**  **1. Patient Positioning and Sterilization**   - **Patient Position**: The patient should be in a supine position with the mouth wide open (the mandibular plane parallel to the ground). - **Sterilization**: The mucosa at the injection site should be sterilized with a 75% alcohol swab.   **2. Injection Site Localization**  **Standard Injection Method**:   - Place the thumb outside the mouth on the posterior border of the mandibular ramus and the index finger inside the mouth on the retromolar pad area. - The injection site is located 3–4 mm lateral to the pterygomandibular fold, approximately at the level of the midpoint connecting the alveolar ridges of the upper and lower jaws (Figure 1).   **3. Needle Insertion Direction and Depth**   - **Needle Selection**: Use a long needle (25–27G, 25–35 mm). - **Insertion Path**:   - Insert the needle horizontally from the contralateral corner of the mouth, directing the tip towards the medial surface of the mandibular ramus.   - Insertion depth: 20–25 mm (15–20 mm for children) until the bone surface (medial plate of the mandible) is contacted. - **Key Techniques**:   - Keep the syringe parallel to the mandibular plane.   - If the bone surface is not contacted, withdraw the needle and adjust the angle before reinserting (to avoid damaging blood vessels by inserting too far posteriorly).   **4. Anesthetic Injection**   - **Drug Selection**: Use 2% lidocaine with 1:100,000 epinephrine or 4% articaine; the adult dose is 1.5–1.8 mL. - **Injection Method**:   - After aspirating to ensure there is no blood, slowly inject the anesthetic.   - When withdrawing the needle to the submucosal level, add an additional 0.5 mL to anesthetize the lingual nerve (to enhance the anesthesia effect on the buccal and lingual sides).   **III. Assessment of Anesthetic Effect**   - **Signs of Success**:   - Numbness of the ipsilateral lower lip and tip of the tongue (indicating successful anesthesia of the inferior alveolar nerve and lingual nerve).   - No pain sensation in the target tooth (test for no response to pulp vitality testing). - **Management of Failure**:   - Check for deviations in the injection site or angle and supplement with additional anesthesia (e.g., buccal nerve infiltration).   **IV. Complications and Management**   - **Hematoma**:   - **Cause**: Puncture of the pterygoid venous plexus or inferior alveolar artery.   - **Management**: Immediate pressure to stop bleeding, ice application, and avoidance of aspirin-like medications. - **Temporary Facial Paralysis**:   - **Cause**: Anesthetic diffusion to branches of the facial nerve.   - **Management**: No intervention required; it will resolve spontaneously within 2–3 hours. - **Nerve Injury**:   - **Prevention**: Avoid forceful needle insertion or repeated punctures.   **V. Key Precautions**   - **Contraindications**:   - Coagulation disorders.   - Uncontrolled local infection. - **Pediatric Adjustments**:   - Reduce insertion depth (approximately 15 mm) and avoid bilateral block simultaneously (to prevent swallowing difficulties). - **Anatomical Variations**:   - For patients with abnormal mandibular foramen locations, use CBCT or ultrasound guidance.   By following these standardized steps and precautions, the inferior alveolar nerve block can be performed safely and effectively, ensuring optimal anesthesia for dental procedures. |
