# Supplementary material for: Exploring the Application Capability of ChatGPT as an Instructor in Skills Education for Dental Medical Students: Randomized Controlled Trial
Source: J Med Internet Res. 2025 May 27;27:e68538. doi: 10.2196/68538 (PMC12152432; doi:10.2196/68538)
Supplement: Multimedia Appendix 2 [file jmir_v27i1e68538_app2.docx]

| **Parameter** | **Specification** |
| --- | --- |
| Sampling Rate | 60Hz |
| Angular Accuracy | 0.1° |
| Binocular Tracking Capability | Yes |
| Head Movement Range | 35*27@60cm |
| Tracking Technology | Corneal Reflection Method, Hybrid dark/bright pupil algorithm and Single-sensor System |
| System Latency | 17 |
| Blink Compensation | 1 frame cycle |
| Interface | USB 2.0 |
| Data Loss Compensation | 250 |

**Reference to aSee A6 eye-tracker.**
