# Supplementary material for: Exploring the Application Capability of ChatGPT as an Instructor in Skills Education for Dental Medical Students: Randomized Controlled Trial
Source: J Med Internet Res. 2025 May 27;27:e68538. doi: 10.2196/68538 (PMC12152432; doi:10.2196/68538)
Supplement: Multimedia Appendix 3 [file jmir_v27i1e68538_app3.docx]

**Knowledge Test**

**1. Which statements are correct regarding periodontal initial therapy?**

A. Gracey 13/14 curettes are used for distal surfaces of posterior teeth
B. During ultrasonic scaling, the working tip should be perpendicular to the tooth surface
C. Bleeding on probing (BOP) is a sensitive indicator of inflammation
D. Occlusal adjustment and mobile tooth fixation should be performed after controlling periodontal inflammation
**Answer:** ACD

**2. Indications for periodontal flap surgery include:**

A. Residual probing depth >5mm with bleeding after initial therapy
B. Complex furcation involvement (Grade II/III)
C. Gingival recession requiring root coverage
D. Intraosseous defects needing bone grafting
**Answer:** ABD

**3. Which of the following statements regarding fixed prosthodontics are correct?**

A. Lingual shoulder margin of full-ceramic crowns should be prepared as 0.8-1mm shallow chamfer
B. In double-cord gingival retraction, place finer cord first followed by thicker cord
C. post–core crown restoration requires at least 2mm residual root filling material
D. When mixing impression materials, add water/powder as needed if ratio is improper
**Answer:** AB

**4. Which of the following statements regarding removable partial dentures (RPD) is incorrect?**

A. Rest seat should be prepared in triangular shape
B. RPI clasp reduces torque on abutments
C. Denture base requires relief in frenum attachment areas
D. Path of insertion must parallel natural tooth long axis
**Answer:** AD

**5. Correct statements about root canal therapy:**

A. Pro Taper instruments feature a variable taper design
B. EDTA gel assists in removing smear layer
C. Optimal obturation requires filling material 0.5-2mm from radiographic apex
D. MTA is indicated for perforation repair
**Answer:** ABCD

**6. Which of the following statements regarding composite resin restorations are correct?**

A. Class V cavities require the preparation of a bevel to increase bonding area
B. Incremental placement with ≤2mm per layer
C. Light-curing duration ≥20 seconds per layer
D. A liner to protect the pulp needed for deep caries
**Answer:** ABCD

**7. What are the key steps in restoring primary molars with stainless steel crowns?**

A. Proximal reduction to decrease tooth diameter
B. Occlusal surface reduction of 1.0-1.5mm
C. Proximal cervical preparation as shallow chamfer
D. Cementation with glass ionomer at crown margin
**Answer:** ABD

**8. Correct management principles for traumatized young permanent teeth:**

A. Immediate replantation with flexible splinting for avulsed teeth
B. Partial pulpotomy for crown fractures with pulp exposure
C. Apexification for pulp necrosis before root completion
D. Immediate repositioning for intruded primary teeth
**Answer:** ABC

**9. Correct procedures for impacted mandibular third molar extraction:**

A. Use of turbine handpiece for tooth sectioning to reduce adjacent tooth resistance
B. Elevator should apply lingual force for tooth displacement
C. Full-thickness mucoperiosteal flap design
D. Interrupted sutures for wound closure
**Answer:** ACD

**10. Which of the following statements regarding basic operative procedures in oral and maxillofacial surgery are incorrect?**

A. Head-neck surgical field disinfection should extend 10cm beyond operative site
B. Electrocautery is the most common hemostatic method
C. Suture knot tails should be trimmed to 3mm in tissues
D. Standard suture spacing: 3-5mm edge distance, 2-3mm needle interval
**Answer:** BCD

**Scoring Criteria:** Each question is worth 10 points, with a total of 100 points. The points for each question are evenly distributed among the correct options. Full marks are awarded for completely correct answers, partial marks are given for partially correct answers, and zero points are awarded if there are any incorrect selections.
